# Serum and urinary biomarkers to predict acute kidney injury in premature infants: a systematic review and meta-analysis of diagnostic accuracy

**DOI:** 10.1007/s40620-022-01307-y

**Published:** 2022-04-06

**Authors:** Jenny Kuo, Lisa K. Akison, Mark D. Chatfield, Peter Trnka, Karen M. Moritz

**Affiliations:** 1 Child Health Research Centre, The University of Queensland, South Brisbane, QLD Australia; 2 School of Biomedical Sciences, The University of Queensland, Sir William MacGregor Building, St Lucia, QLD 4072 Australia; 3 Queensland Child and Adolescent Renal Service, Queensland Children’s Hospital, South Brisbane, QLD Australia

**Keywords:** Paediatric, Serum creatinine, Urinary NGAL, Serum cystatin C

## Abstract

**Background:**

Premature infants are at high risk for acute kidney injury (AKI) and current diagnostic criteria are flawed. The objective of this study was to determine the diagnostic accuracy of urine and serum biomarkers not currently used in routine clinical practice to predict AKI in premature infants.

**Method:**

A systematic review was performed that followed the Preferred Reporting Items for Systematic Reviews and Meta-Analyses of Diagnostic Test Accuracy Studies (PRISMA-DTA). Data were extracted on the diagnostic accuracy of AKI biomarkers using serum creatinine or urine output as the reference standard. Quality and validity were assessed using modified Standards for Reporting Diagnostic Accuracy (STARD) criteria.

**Results:**

We identified 1024 articles, with 15 studies (791 infants) eligible for inclusion. Twenty-seven biomarkers were identified including serum cystatin C and urinary neutrophil gelatinase-associated lipocalin (uNGAL), osteopontin, kidney injury molecule-1, epidermal growth factor, and protein S100-P. However, many were only reported by one study each. A meta-analysis could only be conducted on uNGAL (288 infants from 6 studies) using a hierarchical, random-effects logistic-regression model. uNGAL had a summary sensitivity of 77% (95% CI 58–89%), specificity of 76% (95% CI 57–88%) and AUC-SROC of 0.83 (95% CI 0.80–0.86) for the diagnosis of AKI. By utilising uNGAL, the post-test probability of AKI increased to 52% (95% CI 37–66%) with a positive test and decreased to 9% (95% CI 5–16%) with a negative test if the pre-test probability was 25%.

**Conclusion:**

uNGAL shows promise as a diagnostically accurate biomarker for AKI in premature infants.

**Graphical abstract:**

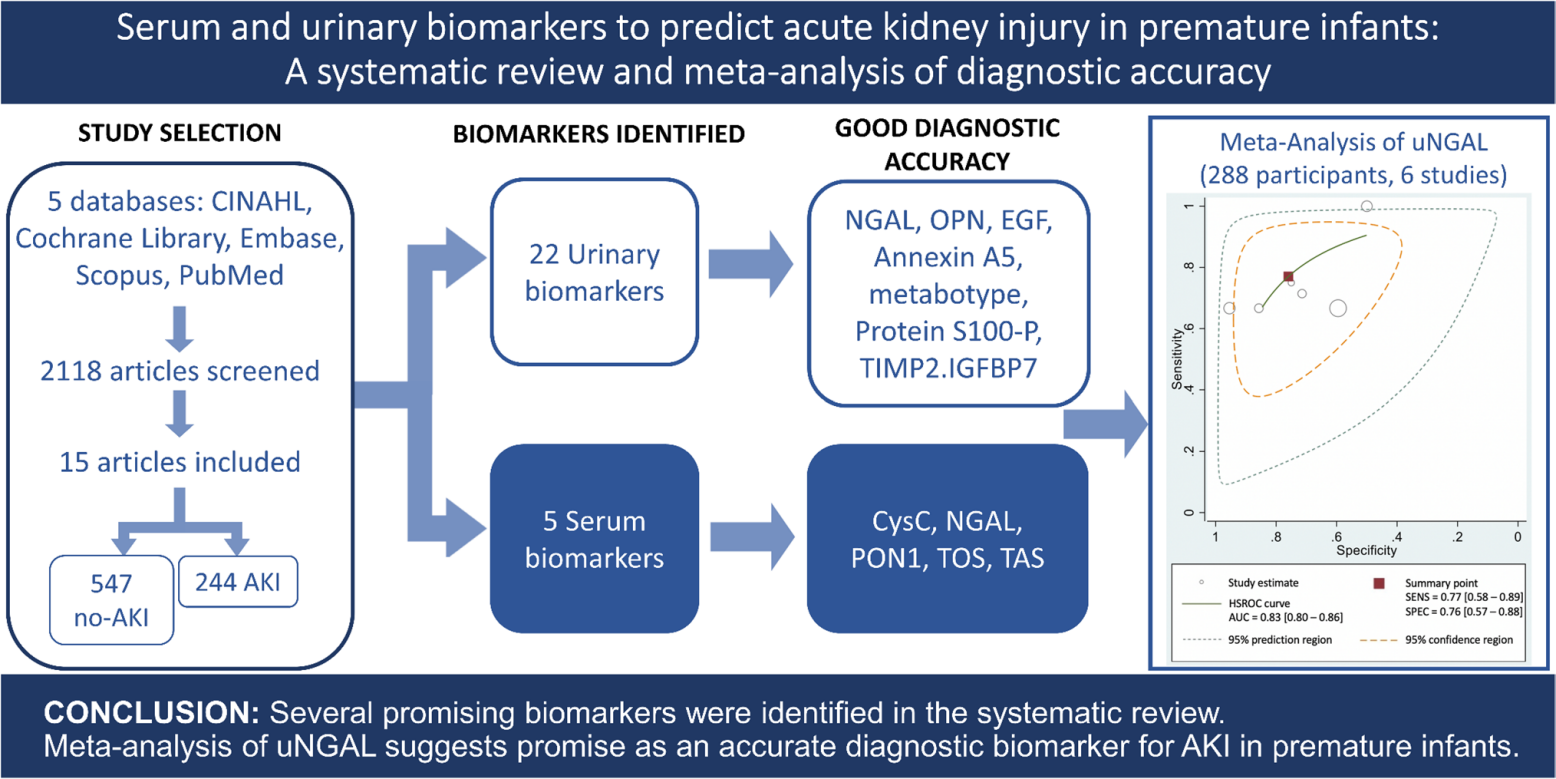

**Supplementary Information:**

The online version contains supplementary material available at 10.1007/s40620-022-01307-y.

## Introduction

Acute kidney injury (AKI) is a significant health issue in premature infants as it correlates with increased morbidity and mortality, and hence, places a significant burden on the health system [1–3]. Currently, AKI is defined by Kidney Disease: Improving Global Outcomes (KDIGO) criteria as an increase in serum creatinine (sCr) above 0.3 mg/dL or 50% from baseline and/or a urine output of less than 0.5 mL/kg/h for at least 6 h [4]. However, these are imprecise parameters of AKI as they are influenced by factors such as age, sex and weight [5]. sCr changes can indicate late consequences of injury because there is a 48 to 72 h delay from the initial renal injury [6, 7]. Significant kidney injury can exist despite minimal changes to sCr levels due to compensatory mechanisms [8, 9]. Neonatal sCr concentrations vary greatly depending on the severity of prematurity and reflect maternal levels up to 72 h post-birth [7, 10, 11]. In neonates born < 32 weeks gestational age, sCr increases after birth and has a peak around day of life 4, which slowly decreases over the first few weeks of life [12–14]. Therefore, sCr is unable to detect AKI in the first few days post-birth. AKI diagnosis based on urine output is also problematic as this is often clinically difficult to monitor in neonates, and non-oliguric renal failure is common in premature infants [7, 15].

An estimated 15 million babies are born prematurely worldwide every year and the rate of premature births is increasing [16]. In the first week of life, the incidence of AKI in premature infants (defined as gestational age < 37 weeks by the World Health Organisation) is reported to be between 12.5 and 39.8% [17–19]. Premature infants are at high risk of an AKI as they have under-developed, and therefore, poorly functioning kidneys. This is typically further complicated by sepsis, nephrotoxic medications and perinatal asphyxia [20–22]. As nephron formation is not completed until 36 weeks of gestation [23], prematurely born infants have a low nephron endowment at birth with subsequent extrauterine nephrogenesis. There is evidence to suggest that perinatal AKI and extrauterine exposures, such as nephrotoxic medications, infections and haemodynamic instability are detrimental to optimal nephrogenesis [24, 25]. Several studies have demonstrated an increased risk for the development of chronic kidney disease (CKD) following an AKI episode in premature children, despite recovery of renal function to pre-morbid estimated glomerular filtration rate (eGFR) levels [26–28]. Three to five years after an initial AKI episode, over 50% of children had at least one sign of CKD [26]. These children are at high risk of progressive CKD and death, and so periodic evaluation following the initial insult is warranted [26, 28].

As traditional markers of AKI are unreliable, several candidates for early detection have recently been investigated. These could facilitate early intervention and validation of therapeutic strategies in clinical practice. Previous reviews regarding the use of urinary and serum biomarkers to diagnose AKI have mainly focused on adult populations [29–32]. While some reviews have included a paediatric population [30, 33–36], none to date have focussed on premature infants whose kidneys would still be undergoing nephrogenesis and functional maturation. Early AKI diagnosis could enable appropriate early management and potentially improve long-term outcomes of this vulnerable population. The current systematic review and meta-analysis aimed to elucidate the diagnostic accuracy of urine and serum biomarkers not currently used in routine clinical practice to predict AKI in premature infants.

## Methods

### Search strategy

This systematic review protocol was registered with the international prospective register of systematic reviews (PROSPERO) as CRD42019122046. The Preferred Reporting Items for Systematic Reviews and Meta-Analyses of Diagnostic Test Accuracy Studies (PRISMA-DTA) statement was followed [37]. A comprehensive, systematic search was undertaken of the following 5 computerised databases: PubMed, EMBASE, CINAHL, Scopus and the Cochrane Library from inception until October 2019. An updated search in August 2020 yielded 1 additional article that fulfilled inclusion criteria [38]. The search strategy included medical subject heading (MeSH) terms and synonymous terms covering the age group of interest (e.g. infant/paediatric/neonate/newborn), AKI and biomarker (see Online Resource 1 1 for full search strategy). Reference lists of included studies and on-topic review articles were manually searched for additional studies not identified by the search strategy. Conference abstracts were excluded but cross-referenced with full publications by the same authors. After duplicate articles were removed, the title and abstract of all retrieved articles were screened for eligibility. Full publications were independently reviewed against an inclusion/exclusion criteria by two authors (JK and LA). Conflicting viewpoints were resolved via discussion until a consensus was reached.

### Study selection criteria

The a priori inclusion criteria were: (1) infants (< 1 years old); (2) premature birth (gestational age < 37 weeks); (3) biomarker measurement in urine, serum, blood or plasma; (4) diagnostic accuracy measures of a biomarker; (5) clear definition and outcome of AKI; (6) human studies; and (7) published in English. Criteria for exclusion were: (1) no reported diagnostic accuracy measures for any biomarkers; (2) no reported AKI; and (3) conference abstract, PhD dissertation, review article or other editorial.

### Study quality assessment

Methodological quality and validity of studies were assessed using a modified 10-itemchecklist of the Standards for Reporting Diagnostic Accuracy (STARD) criteria adapted from Coca et al. [29] (see Online Resource 2). Articles with a quality assessment score ≤ 6 were excluded.

### Data extraction and synthesis

Information on the study setting, biomarkers (including type, measurement/timing and threshold for diagnosis), number and gestational age of participants, and criteria for confirming AKI diagnosis (see Online Resource 3 for definitions), were extracted and summarised for the included studies by JK. For each biomarker, data on sensitivity/specificity, area under the receiver operating characteristic curve (AUROC), and likelihood ratios (LRs) were extracted. Biomarkers with AUROC values > 0.70 are defined as a good measure of diagnostic accuracy [39]. Biomarker cut-offs for AKI diagnosis were extracted. An attempt was made to contact authors for raw data if information was not provided.

### Meta-analysis

Meta-analysis was conducted using Stata 16 (StataCorp LLC, College Station TX) and the user-written Stata metandi package for analysis of diagnostic accuracy using hierarchical logistic regression [40, 41]. Due to limitations of metandi, a meta-analysis could only be completed for biomarkers with four or more included studies (i.e. urine neutrophil gelatinase-associated lipocalin [uNGAL]). 2 × 2 contingency tables were estimated using published numbers of participants, sensitivity/specificity and/or AUROC data. Where studies published multiple sets of data for one biomarker at varied time points, only one dataset from each paper was included in the meta-analysis. This was pre-determined to be data from 1 day prior to AKI diagnosis and day of life 1 for consistency between studies. Summary sensitivities, specificities, diagnostic odds ratios, LRs and a summary receiver operator characteristic curve (SROC) with 95% confidence and prediction intervals for the diagnostic accuracy of uNGAL were determined. The corresponding area under the SROC curve (AUC-SROC) was estimated. A Fagan nomogram (Bayes nomogram) is a clinically useful tool that objectively quantifies the post-test probability of a patient having a condition, in this case, the probability of an infant having an AKI after a uNGAL test. This nomogram was constructed with the Stata midas package. A pre-test probability of 25% was used, which is the average reported prevalence of AKI in premature infants [19, 42, 43].

## Results

### Search results

**Fig. 1:**
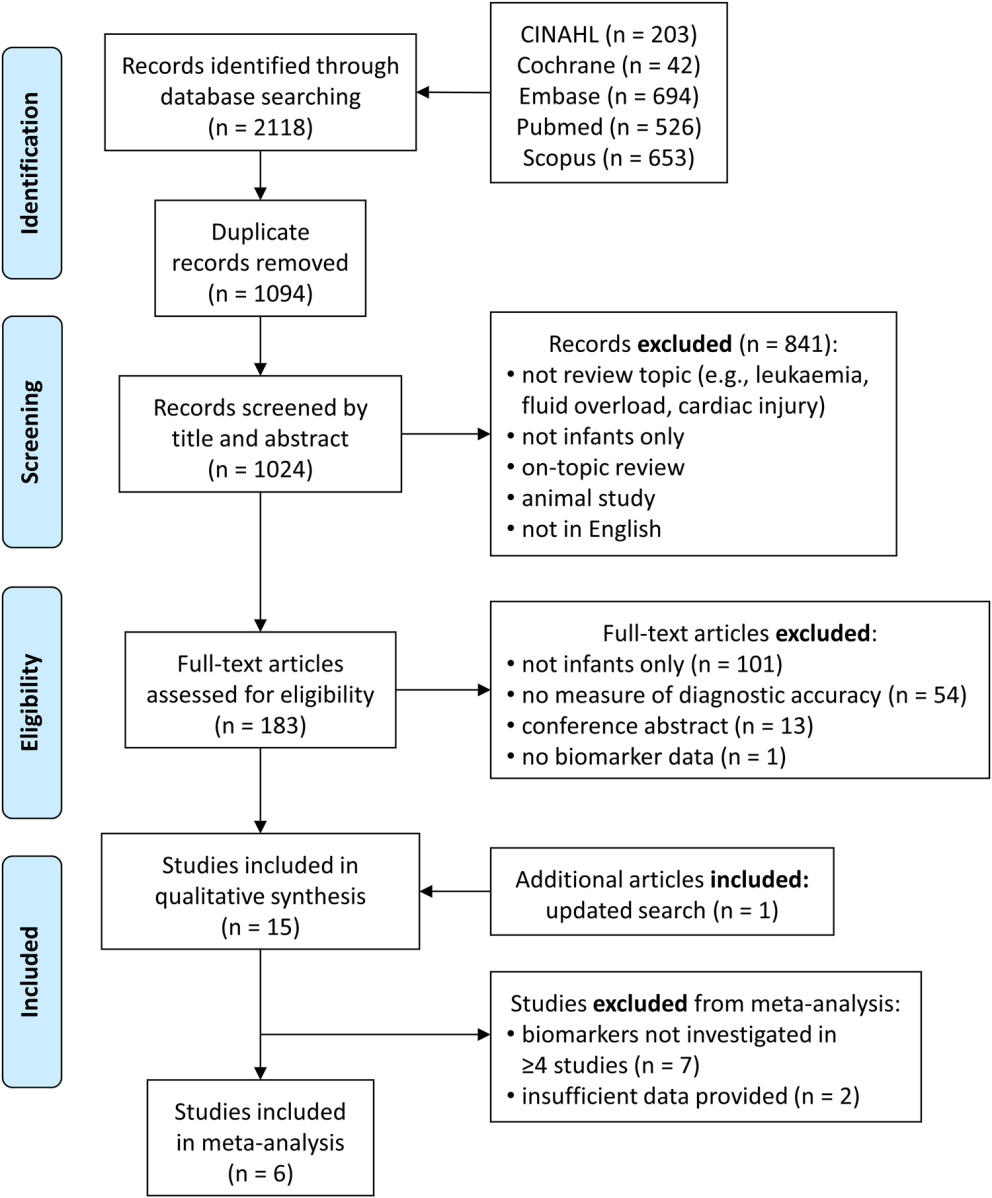
Flow diagram of the literature search strategy and study selection process (based on the PRISMA-DTA statement [37]). See text for a full description of exclusion criteria. The original search was completed in October 2019, with an updated search in August 2020. uNGAL = urinary neutrophil gelatinase-associated lipocalin

The search identified 2118 articles (Fig. 1). After duplicates were removed, the title and abstracts of 1024 citations were reviewed against the selection criteria. Once 841 studies were excluded, the full texts of 183 citations were screened against inclusion/exclusion criteria, with 15 studies meeting inclusion criteria (Fig. 1) [38, 44–57]. The major reasons for exclusion were study populations in children greater than 1 year-old, non-premature infants, no AKI outcome, no biomarker diagnosis of AKI, and non-English publications. Eight studies [44, 47, 48, 50–52, 57] investigated uNGAL but only 6 of these [38, 44, 47, 48, 50, 51] provided sufficient data to construct 2 × 2 contingency tables to be included in the meta-analysis. One study [58] reporting on uNGAL was not included as the data presented were essentially a repeat of the data included from Tabel et al. [48].

### Assessment of study quality

No studies were excluded, as all had a score of ≥ 7 out of a possible score of 11 (see Online Resource 4). While participant sampling, data collection and rationale for reference standards and biomarkers were similar in all studies, only 8 studies fully stated the specifications of biomarker measurements and analysis [46, 47, 49, 51–54, 57]. Reference standards for clinical AKI diagnosis were based on sCr only and were used to interpret index test results. Only 2 studies [47, 49] reported blinding of readers to the index test and reference standard. None stated whether they had a representative distribution of AKI severity, with most considering all neonates with AKI as one group. Some studies did not have participants representing the full spectrum of severity (i.e. mild, moderate or severe).

### Description of study characteristics

Study characteristics are presented in Table 1, including the number of participants, gestational age, criteria used for AKI diagnosis, biomarker measurement method, determination of biomarker threshold and quality assessment score. All studies investigated biomarkers prospectively and were case–control, cross-sectional or cohort studies, with patient eligibility criteria clearly pre-defined. Studies were published between 2011 and 2020 from 7 countries (Egypt, USA, Turkey, Serbia, Greece, South Korea and Germany). All but one were single-centre studies [52], where participants were recruited from two hospitals. Only premature infants were enrolled but many had various medical conditions including respiratory distress syndrome, perinatal asphyxia, very low birth weight or patent ductus arteriosus. Amongst the studies, there were differences in gestational age [48, 52, 56] and weight [44, 48, 51–53] but not gender between AKI and no-AKI participants. All but one of the studies were conducted in neonatal intensive care units (NICU) [55], which did not specify the setting. A total of 27 biomarkers were investigated (Table 1). Of these, there were 5 serum and 22 urinary biomarkers, with the most common being uNGAL (8 studies). Urinary cystatin C (uCysC), kidney injury molecule 1, beta-2 microglobulin and osteopontin and serum cystatin C (sCysC) were each investigated in three studies. Urinary albumin and epidermal growth factor were investigated in two studies each. Remaining biomarkers were each investigated in one study. All samples were stored at − 70 °C prior to biomarker measurement using an assortment of assays (see Table 1 for details). AKI diagnostic criteria varied between studies but were relatively consistent in terms of sCr thresholds (Table 1). Eleven studies determined biomarker cut-off values for AKI diagnosis.

**Table 1:** Characteristics and participant details of included studies

Study^a^	Biomarker(s)	Participants (n)		GA (wks)	AKI Diagnosis^b^ (criteria or change in sCr^h^)	Measurement method	Determination of threshold for diagnosis	Quality score (max 11)
		No-AKI	AKI					
Urine								
Askenazi et al. [44]	B2M, CysC, IL-18, KIM-1, NGAL, OPN	21	9	< 31	AKIN^c^	ECLIA	N/A	8
Askenazi et al. [50]	α-GST, Albumin, B2M, Calbindin, Clusterin, CysC, EGF, KIM-1, NGAL, OPN, Osteo-activin, TFF3, VEGF	85	28	< 31	Neonatal KDIGO (2014)	ECLIA	Logistic regression adjusted for gestational age to calculate ROC	7
Genc et al. [46]	KIM-1	30	18	26–34	> 1.3 mg/dL after 60 h of life; Stage I KDIGO	ELISA	ROC curve analysis	9
Hanna et al. [55]	Albumin, B2M, CysC, EGF, NGAL, OPN, UMOD	20	25	< 32	Neonatal KDIGO (2012)^d^	ECLIA	N/A	8
Jung et al. [38]	NGAL, CILP-2, 6-PGLS, Annexin A5, Galectin 3, Protein S100-P	14	14	< 32	AKIN or persistent increase in sCr to ≥ 1.7 mg/dL for 3 days after birth	ELISA	ROC curve analysis	8
Mercier et al. [55]	Metabolites^e^	20	20	≤ 31	KDIGO	NMR spectroscopy	Logistic regression using calculated pH and metabotype	8
Parravicini et al. [52]	NGAL	78	13	< 30	Neonatal KDIGO^f^	Immunoblot	> 50 ng/mL	9
Sarafidis et al. [47]	NGAL	11	11	27–32	Neonatal KDIGO (2012)^d^	ELISA	ROC curve analysis	10
Tabel et al. [48]	NGAL	44	6	28–34	Day1: > 1.5 mg/dL for > 48 h while mother has normal renal function urine output < 0.5 mL/kg/h for 8 h and/or sCr rising to > 0.3 mg/dL or more from baseline within 24 h	ELISA	ROC curve analysis	8
Waldherr et al. [57]	Calprotectin, KIM-1, NGAL, [TIMP-2].[IGFBP7]	11^g^	3	≤ 31	Neonatal KDIGO (2014)	CMIA and ELISA	N/A	8
Serum								
Abdelaal et al. [53]	CysC	36^g^	24	28–36	Neonatal KDIGO (2012)^d^	PENIA	ROC curve analysis	9
El-Gammacy et al. [56]	CysC	37^g^	13	< 34	pRIFLE	ELISA	ROC curve analysis	8
Elmas et al. [45]	CysC	22^g^	6	27–32	> 1.5 mg/dL on first 3 days of life and/or pRIFLE	PENIA	ROC curve analysis	8
Ivanisevic et al. [54]	PON1, TAS, TOS	45	19	32–36	≥ 26.5 µmol/L difference between Day 1 and 3	Spectrophoto-meter	N/A	9
Pejovic et al. [49]	NGAL	73	35	< 37	Neonatal KDIGO (2012)^d^	ELISA	ROC curve analysis	10

Biomarkers were tested in urine (n = 10 studies) or serum (n = 5 studies)

6-PGLS: 6-phosphogluconolactonase; α-GST: α-glutathione S-transferase; AKI: acute kidney injury; AKIN: Acute Kidney Injury Network [66]; B2M: beta-2 microglobulin; CILP-2: cartilage intermediate layer protein 2; CMIA: chemiluminescent microparticle immunoassay; CysC: cystatin C; ECLIA: electrochemiluminescence immunoassay; EGF: epidermal growth factor; ELISA: enzyme-linked immunosorbent assay; GA: gestational age; h: hour; IL-18: interleukin 18; KDIGO: Kidney Disease: Improving Global Outcomes [4, 11, 67]; KIM-1: kidney injury molecule-1; Max: maximum; N/A: not applicable; NGAL: neutrophil gelatinase-associated lipocalin; NICU: neonatal intensive care unit; NMR: nuclear magnetic resonance; OPN: osteopontin; PENIA: particle-enhanced nephelometric immunoassay; PON1: paraoxonase 1; pRIFLE: pediatric Risk, Injury, Failure, Loss, End Stage Renal Disease [68]; ROC: receiver operator characteristic; sCr: serum creatinine; TAS: total antioxidant status; TFF3: trefoil factor 3; [TIMP-2].[IGFBP7]: tissue inhibitor of metalloproteinase-2 with insulin-like growth factor binding protein 7; TOS: total oxidant status; UMOD: uromodulin; VEGF: vascular endothelial growth factor; Wks: weeks

^a^All studies recruited participants in the neonatal intensive care unit, with the exception of Mercier et al. [55] which did not specify. All study participants were premature infants

^b^Refer to Online Resource 3 for details of each AKI criteria

^c^Only stage I of the AKIN criteria was used

^d^Neonatal KDIGO described by Jetton and Askenazi [11] does not include a urine output criteria compared to neonatal KDIGO described by Jetton and Askenazi [67]. Each SCr is compared to the lowest previous SCr value

^e^Metabolites that differentiated AKI from no-AKI profiles were: 1,7-dimethylxanthine, benzoate, cholate, choline, ippurate, homovanillate, lysine acetate, myo-inositol, N-acetyltyrosine, sugars, tyrosine, xanthine

^f^ ≥ 0.3 mg/dL rise from baseline for at least 2 sCr values within 48 h

^g^Additional participants were included in the paper as a control group but data on AKI biomarkers only in this subsample

^h^Conversion factors for units: serum creatinine in mg/dL to μmol/L, × 88.4

### Data synthesis

Data extracted from studies are detailed in Table 2, including the timing of measurements, biomarker threshold for diagnosis, sensitivity, specificity, AUROC, and positive and negative likelihood ratios. Specific information for serum and urinary biomarkers is summarised below.

**Table 2:** Diagnostic methods and sensitivity/specificity of tested biomarkers for predicting acute kidney injury (AKI)

Biomarker	Study	Timing of measurements (day = day of life)	Biomarker threshold for diagnosis	Sensitivity/specificity (%)	AUROC	LR+/LR−
Urine						
NGAL	Askenazi et al. [44]	Daily until day 6	NR	66.7/85.7	0.80	NR
	Askenazi et al. [50]	Daily until day 4	450 ng/mL	NR	0.63	NR
	Hanna et al. [55]	1 day prior to stage I AKI Dx	NR	100/50	0.91	NR
		1 day prior to stage II/III AKI Dx	NR	NR	0.92	NR
	Jung et al. [38]	1st urine on day 1	42.15 ng/mL	71/71	0.75	NR
	Parravicini et al. [52]	Daily until 32 wk postmenstrual age, discharge or death	> 50 ng/mL	61/63	0.62	1.65/0.62
	Sarafidis et al. [47]	Day 0 (AKI diagnosis)	19.39 ng/mL	54.5/90.9	0.744	NR
		1 day prior to stage I AKI Dx^a^	18.95 ng/mL	75/75	0.703	NR
		2 days prior to stage I AKI Dx^a^	19.99 ng/mL	44.4/100	0.736	NR
	Tabel et al. [48]	Day 1	38.3 ng/mL	66.7/95.5	0.85	14.8/1.02
		Day 7	30.6 ng/mL	50/91	NR	5.4/1.04
	Waldherr et al. [57]	Before indomethacin	NR	NR	0.13	NR
		6 h after indomethacin	NR	NR	0.27	NR
		12 h after indomethacin	NR	NR	0.39	NR
NGAL/Cr	Askenazi et al. [50]	Daily until day 4	4.8 × 10^6^ pg/mL	NR	0.69	NR
CysC	Askenazi et al. [44]	Daily until day 6	NR	NR	0.73	NR
	Askenazi et al. [50]	Daily until day 4	1.26 × 10^6^ pg/mL	NR	0.65	NR
	Hanna et al. [55]	1 day prior to stage I AKI Dx	NR	NR	0.79	NR
		1 day prior to stage II/III AKI Dx	NR	NR	0.82	NR
CysC/Cr	Askenazi et al. [50]	Daily until Day 4	1.1 × 10^7^ pg/mL	NR	0.69	NR
KIM-1	Askenazi et al. [44]	Daily until day 6	NR	NR	0.50	NR
	Genc et al. [46]	Days 1, 3, 7^b^	↑0.5 ng/mg Cr from DOL 1–3	73.3/76.9	0.791	78.6/71.4
	Waldherr et al. [57]	Before indomethacin	NR	NR	0.33	NR
		6 h after indomethacin	NR	NR	0.50	NR
		12 h after indomethacin	NR	NR	0.70	NR
OPN	Askenazi et al. [44]	Daily until day 6	NR	NR	0.83	NR
	Askenazi et al. [50]	Daily until day 4	7.12 × 10^5^ pg/mL	NR	0.65	NR
	Hanna et al. [55]	1 day prior to stage I AKI Dx	NR	NR	0.8	NR
		1 day prior to stage II/III AKI Dx	NR	NR	0.84	NR
OPN/Cr	Askenazi et al. [50]	Daily until day 4	5.7 × 10^6^ pg/mL	NR	0.71	NR
B2M	Askenazi et al. [44]	Daily until day 6	NR	NR	0.66	NR
	Hanna et al. [55]	1 day prior to stage I AKI Dx	NR	NR	0.6	NR
		1 day prior to stage II/III AKI Dx	NR	NR	0.49	NR
B2M/Cr	Askenazi et al. [50]	Daily until day 4	1.2 × 10^8^ pg/mL	NR	0.67	NR
Albumin	Askenazi et al. [50]	Daily until day 4	3.69 × 10^7^ pg/mL	NR	0.62	NR
	Hanna et al. [55]	1 day prior to stage I AKI Dx	NR	NR	0.66	NR
		1 day prior to stage II/III AKI Dx	NR	NR	0.59	NR
Albumin/Cr	Askenazi et al. [50]	Daily until day 4	3.14 × 10^8^ pg/mL	NR	0.67	NR
EGF	Askenazi et al. [50]	Daily until day 4	5.9 × 10^2^ pg/mL^c^	NR	0.68	NR
	Hanna et al. [55]	1 day prior to stage I AKI Dx	NR	NR	0.97	NR
		1 day prior to stage II/III AKI Dx	NR	NR	0.86	NR
UMOD	Askenazi et al. [50]	Daily until day 4	6.7 × 10^5^ pg/mL^c^	NR	0.71	NR
	Hanna et al. [55]	1 day prior to stage I AKI Dx	NR	NR	0.87	NR
		1 day prior to stage II/III AKI Dx	NR	NR	0.85	NR
UMOD/Cr	Askenazi et al. [50]	Daily until day 4	6.9 × 10^6^ pg/mL	NR	0.64	NR
6-PGLS	Jung et al. [38]	1^st^ urine on day 1	NR	NR	0.67	NR
α-GST	Askenazi et al. [50]	Daily until day 4	4.33 × 10^2^ pg/mL	NR	0.68	NR
α-GST/Cr	Askenazi et al. [50]	Daily until day 4	4.5 × 10^3^ pg/mL	NR	0.71	NR
Annexin A5	Jung et al. [38]	1^st^ urine on day 1	409.86 pg/mL	85/71	0.882	NR
Clusterin	Askenazi et al. [50]	Daily until day 4	3.1 × 10^5^ pg/mL	NR	0.62	NR
Clusterin/Cr	Askenazi et al. [50]	Daily until day 4	2.4 × 10^6^ pg/mL	NR	0.68	NR
Calprotectin	Waldherr et al. [57]	Before indomethacin	NR	NR	0.56	NR
		6 h after indomethacin	NR	NR	0.47	NR
		12 h after indomethacin	NR	NR	0.06	NR
Galectin 3	Jung et al. [38]	1^st^ urine on day 1	NR	NR	0.472	NR
IL18	Askenazi et al. [44]	Daily until day 6	NR	NR	0.60	NR
Metabolites^d^	Mercier et al. [55]	Day 2	NR	NR	0.93	NR
Protein S100-P	Jung et al. [38]	1st urine on day 1	110.70 pg/mL	83/64	0.748	NR
[TIMP-2]. [IGFBP7]	Waldherr et al. [57]	Before indomethacin	NR	NR	0.27	NR
		6 h after indomethacin	NR	NR	0.80	NR
		12 h after indomethacin	NR	NR	1.00	NR
VEGF/Cr	Askenazi et al. [50]	Daily until day 4	1.2 × 10^4^ pg/mL	NR	0.64	NR
Serum						
CysC	Abdelaal et al. [53]	Day 3	1.28 mg/L	100/83.3	0.97	NR
	El-Gammacy et al. [56]	Day 3	1.3 mg/L	92.3/96	0.926	NR
	Elmas et al. [45]	Day 3	≥ 1.62 mg/L	16/82	0.88	9.2/1.02
PON1, TOS and TAS^e^	Ivanisevic et al. [54]	Day 1–2 h	NR	NR	0.615	NR
		Day 1–4 h	NR	NR	0.900	NR
		Day 1–6 h	NR	NR	0.857	NR
		Day 3	NR	NR	0.583	NR
		Day 7	NR	NR	0.893	NR
NGAL	Pejovic et al. [49]^f^	Day 1–2 h	84.9 ng/mL	69.0/71.9	0.72	2.45/0.43
		Day 1–4 h	89.4 ng/mL	65.8/74.3	0.72	2.56/0.46
		Day 1–6 h	54.2 ng/mL	88.9/44.1	0.68	1.59/0.25
		Day 3	106.5 ng/mL	45.8/82.9	0.63	2.67/0.65
		Day 7	38.2 ng/mL	88.9/34.3	0.59	1.35/0.32

Biomarkers were tested in urine (n = 10 studies) or serum (n = 5 studies)

6-PGLS: 6-phosphogluconolactonase; α-GST: α-glutathione S-transferase; AKI: acute kidney injury; AUROC: area under the receiver operating characteristic curve; B2M: beta-2 microglobulin; Cr: creatinine; CysC: cystatin C; Dx: diagnosis; EGF: epidermal growth factor; h: hour; IL-18: interleukin 18; KIM-1: kidney injury molecule-1; LR + /LR-: positive likelihood ratio/negative likelihood ratio; n: participants; NGAL: neutrophil gelatinase-associated lipocalin; NICU: neonatal intensive care unit; NR: not reported; OPN: osteopontin; PDA: patent ductus arteriosus; PON1: paraoxonase 1; RDS: respiratory distress syndrome; TAS: total oxidant status; [TIMP-2.IGFBP7]: tissue inhibitor of metalloproteinase-2 and insulin-like growth factor binding protein 7; TOS: total antioxidant status; UMOD: uromodulin; VEGF: vascular endothelial growth factor; VLBW: very low birth weight; Wk: week

^a^Not explicitly stated to be stage I AKI in study but it is assumed to be stage I AKI

^b^Timing of measurements were on DOL 1, 3 and 7 but diagnostic accuracy measures are for from days 1 to 3

^c^Values are minimum urinary biomarker levels

^d^Metabolites that differentiated AKI from no-AKI profiles were: 1,7-dimethylxanthine, benzoate, cholate, choline, hippurate, homovanillate, lysine acetate, myo-inositol, N-acetyltyrosine, sugars, tyrosine, xanthine. Hippurate and homovanillate were found to differentiate AKI from no-AKI with statistical significance

^e^AUROC values were only reported as a combination of PON1, TOS and TAS

^f^Number of participants included in the analysis varied with measurement times. DOL 1: 2 h (n = 103), DOL 1: 4 h (n = 108), DOL 1: 6 h (n = 106), DOL 3 (n = 107), DOL 7 (n = 107)

#### Serum biomarkers

sCysC values were significantly raised in AKI compared to no-AKI patients in all 3 studies, with AUROC values ranging from 0.88 to 0.97 [45, 53, 56], suggesting sCysC is a very good to excellent measure of diagnostic accuracy. Serum NGAL (sNGAL) was significantly elevated in AKI compared with no-AKI participants on day of life 1 at 2, 4 and 6 h after NICU admission. The combination of serum paraoxonase 1, total oxidant status and total antioxidant status showed good diagnostic accuracy on day of life 1 at 4 h and 6 h and day of life 7. However, these biomarkers were each only investigated in a single study (Table 2).

#### Urinary biomarkers

uNGAL, a marker of renal tubular inflammation, was significantly increased in AKI compared to no-AKI controls in all but one study (Table 2) [38, 44, 47, 48, 50–52, 57]. The only exception was Waldherr et al. [57] in which uNGAL AUROC values ranged from 0.13 to 0.39, with AUROC values increasing after indomethacin administration.

Urinary osteopontin, epidermal growth factor and uromodulin appeared to be of very good diagnostic value, with the majority of studies reporting AUROC > 0.8. uCysC was significantly elevated in AKI compared to no-AKI patients, with AUROC ranging from 0.65 to 0.82 in all three studies where it was measured (Table 2) [44, 50, 51]. [TIMP-2].[IGFBP7] (tissue inhibitor of metalloproteinases-2 and insulin-like growth factor binding protein 7) was only explored in one study but appeared to be of significant diagnostic value at 6 h and 12 h after indomethacin administration, with AUROCs of 0.80 and 1.00 respectively. Annexin A5 (AUROC = 0.88) and protein S100-P (AUROC = 0.75) also demonstrated good diagnostic accuracy but were only investigated in one study [38]. The metabotypes investigated by Mercier et al. [55], especially hippurate and homovanillate, showed significant differences between AKI and no-AKI groups on day of life 2 (AUROC = 0.93). Urinary kidney injury molecule 1 showed unclear diagnostic accuracy value but appeared to be significantly higher in AKI compared to no-AKI from days of life 1 to 3 and 12 h after indomethacin treatment. Urinary albumin, beta-2 microglobulin, interleukin 18, α-glutathione S-transferase, clusterin, calprotectin, Galectin 3 and 6-phosphogluconolactonase did not appear to be of any significant value in diagnosing AKI (AUROC values < 0.7), although data were derived from a single study for most of these biomarkers (Table 2).

Hanna et al. [51] differentiated the AKI population into 2 groups according to neonatal KDIGO criteria (stage I and stage II/III). uNGAL (0.92 vs 0.91), uCysC (0.82 vs 0.79), urinary uromodulin (0.85 vs 0.87) and urinary osteopontin (0.84 vs 0.80) had relatively similar AUROC for stage II/III AKI and stage I AKI. However, AUROC for urinary beta-2 microglobulin(0.49 vs 0.6), albumin (0.59 vs 0.66), and epidermal growth factor (0.86 vs 0.97) were lower in stage II/III AKI than stage I AKI.

### Meta-analysis of uNGAL diagnostic accuracy for detecting AKI

**Fig. 2:**
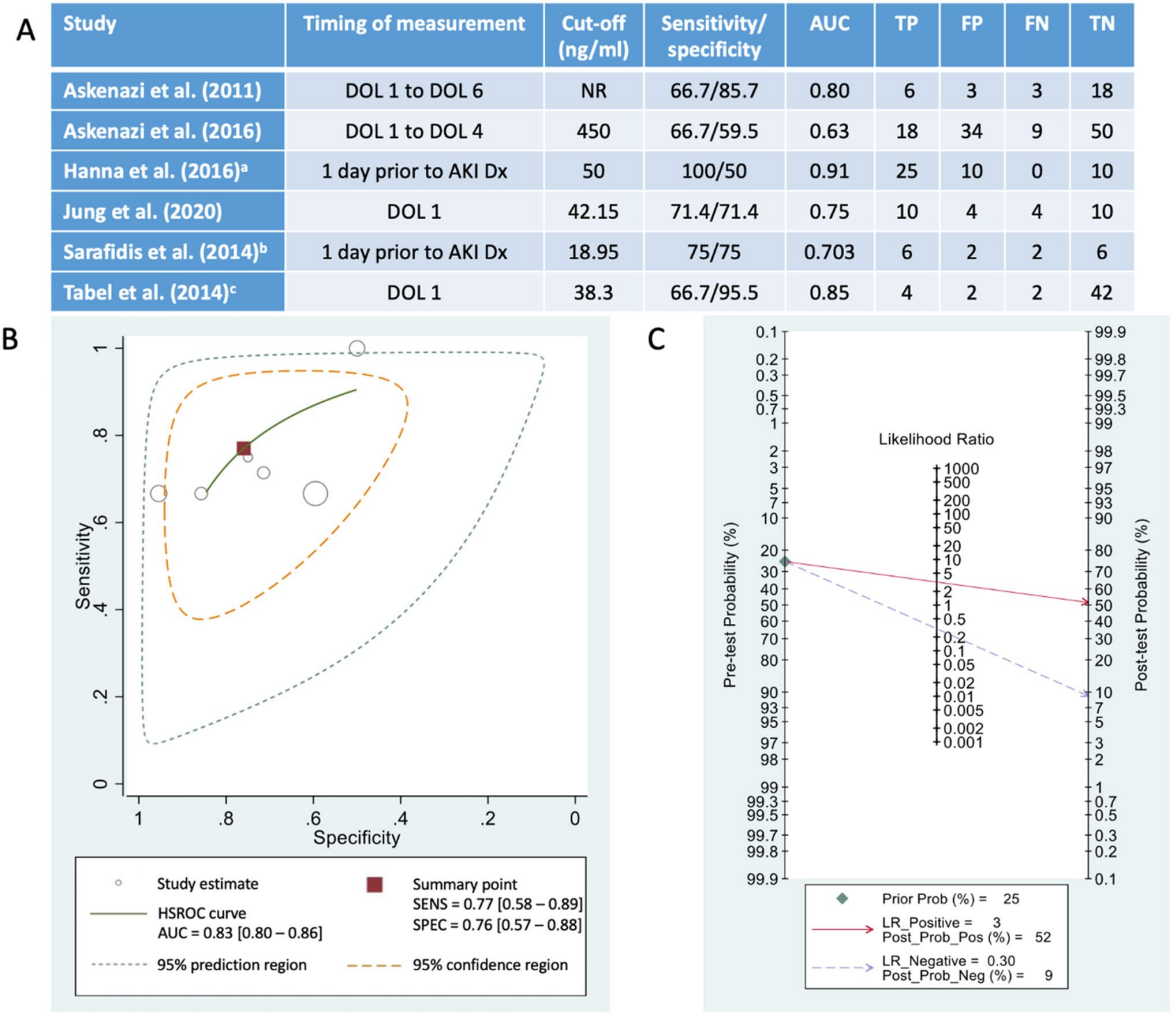
Meta-analysis of diagnostic accuracy of urinary neutrophil gelatinase-associated lipocalin (uNGAL) in predicting AKI in premature infants. Analysis was conducted using six included studies measuring uNGAL [44, 47, 48, 50, 51]. **A** 2 × 2 contingency table data used for each of the studies included in the meta-analysis. AUC: area under the curve; Dx: diagnosis; FN: false negative; FP: false positive; TN: true negative; TP: true positive; **B** Hierarchical summary receiver operator characteristic curve (HSROC) with 95% confidence and prediction regions. Calculated in Stata using the metandi and midas commands. Grey circles represent each of the six studies included in the analysis, with size indicating the sample size. The red square represents the overall estimate of sensitivity (SENS), specificity (SPEC). **C** Fagan plot (Bayes nomogram) created using the midas command within STATA. ^a^Estimated from measurement at 1 day prior to stage I acute kidney injury (AKI) diagnosis. Data for contingency table obtained from Figure 1e in Hanna et al. [51]. Biomarker cut-off as per Parravicini et al. [52]. ^b^Estimated from measurement at 1 day prior to AKI diagnosis (stage not specified). ^c^Estimated from measurement at day of life (DOL) 1

The meta-analysis included 288 participants [no-AKI (n = 195) and AKI (n = 93)] from six studies (Fig. 2A). uNGAL had a summary sensitivity of 77% (95% CI 58–89%), specificity of 76% (95% CI 57–88%) and diagnostic odds ratio of 11 (95% CI 4–28) for AKI diagnosis (Fig. 2B). The SROC curve showed an AUROC of 0.83 (95% CI 0.80–0.86), which suggests that uNGAL has a good diagnostic accuracy for AKI in premature infants (Fig. 2B). Positive and negative LRs were 3.2 (95% CI 1.8–5.8) and 0.30 (95% CI 0.16–0.57). Fagan’s nomogram is a tool that illustrates the post-test probability of a premature infant having AKI. When the pre-test probability of AKI is 25%, Fagan’s nomogram shows that a high uNGAL result increases the post-test probability of AKI to 52% (95% CI 37–66%) while a low uNGAL result decreases the post-test probability to 9% (95% CI 5–16%) (Fig. 2C).

## Discussion

This systematic review and meta-analysis of biomarkers of AKI in premature babies suggests that sCysC and uNGAL have the highest diagnostic accuracy in this clinical setting. Our review highlights that a range of urinary and serum biomarkers have been tested but only in a limited number of small studies involving premature babies.

Of all the serum biomarkers investigated, sCysC had the highest diagnostic accuracy, with AUROC values between 0.88 and 0.97. Cystatin C is a cysteine protease inhibitor which is produced at a constant rate and exclusively excreted with no reabsorption by the renal tubules [59]. Unlike serum creatinine, sCysC is not influenced by sex, age or muscle mass and does not cross the placenta, so concentrations in the early postnatal period are reflective of early neonatal kidney function, rather than maternal concentrations [59–62]. Serum NGAL and the combination of serum paraoxonase 1, total oxidant status and total antioxidant status were only of significant diagnostic value at certain time-points. While sCysC performed the best in terms of diagnostic accuracy, and two studies reported high sensitivity (92.3%, 100%), one study reported a sensitivity of only 16% to predict AKI. It also relies on blood sampling during the neonatal period which can be problematic [63]. Although microsampling techniques such as dried blood spots can be used with ultrasensitive analytical methods such as liquid-chromatography with tandem mass-spectrometry, thereby dramatically reducing the blood volume required, urinary biomarkers are still generally considered more attractive in this clinical setting because of their non-invasive nature.

This systematic review found that urinary NGAL, osteopontin, epidermal growth factor, uromodulin, CysC, [TIMP-2].[IGFBP7], annexin A5, protein S100-P and metabotype, generally provided good diagnostic value with AUROC > 0.7. All other urinary biomarkers had AUROCs below 0.7 and would be less likely to reliably diagnose AKI in premature infants, although most were only investigated in one or two studies. From the meta-analysis, uNGAL showed good diagnostic accuracy for AKI in premature infants with a high summary sensitivity, specificity and AUROC. In the SROC, the confidence and prediction regions were large, which could be attributed to having a small number of included studies and small sample sizes. Other factors that may influence this include variable gestational age ranges, measurement methods and timing, and clinical settings.

### Limitations

Although studies included in this systematic review were generally of high quality in terms of reporting, many biomarkers were only investigated in one or two studies or had small numbers of infants developing AKI (i.e. < 20 in 10 studies). Waldherr et al. [57] in particular only had 3 of 14 participants developing AKI, with potential implications for power and statistical significance of the results. From the quality assessment, the lack of blinding of readers to AKI diagnosis or a representative distribution of disorder severity may increase the risk of bias. In a few studies, there were statistically significant differences in gestational age [48, 52, 56] and weight [44, 48, 51–53] between infants with and without AKI, which may influence biomarker concentrations and skew diagnostic accuracy.

While the reference standard for AKI diagnosis was based on serum creatinine for all studies, there were variations in criteria between the studies. AKI criteria used were pRIFLE, KDIGO, modified KDIGO/new proposed neonatal AKI [11] or adapted own definitions based on pre-existing criteria. Additionally, studies had different timings for collection of urine for biomarker measurements. For example, biomarkers may have been measured daily until a certain time point, in relation to AKI diagnosis (e.g. 1 day prior), or in relation to medication administration. AKI and non-AKI groups were then determined retrospectively based on serum creatine. The exact timing of kidney injury is unclear, and this poses a limitation in determining time-related diagnostic performance.

Several studies did not report cut-off values for biomarkers. For example, only 6 out of 8 studies investigating uNGAL reported diagnostic cut-offs and only one study had a pre-defined cut-off of 50 ng/mL. Cut-off thresholds for uNGAL varied, with one study reporting an unexpectedly high threshold of 450 ng/mL (compared to 19–50 ng/mL in other studies). Studies likely used cut-offs that optimised AUROC values which may have resulted in an overestimation of diagnostic accuracy. All these factors are likely to result in discrepancies between studies. Future studies should assess these promising biomarkers taking these factors into account, and should consider standardising cut-off values and time points to validate their utility as a good measure of diagnostic accuracy. Although all studies report AUROC, inclusion of 2 × 2 tables and sensitivity and specificity values would also be useful for consistency and ease of interpretation. Given 2 × 2 tables were not provided by the included studies, these had to be calculated from published data in order to conduct the meta-analysis. Additionally, we were unable to determine an ideal diagnostic cut-off for uNGAL, as the raw data was not provided and could not be obtained from study authors.

No studies have serial measurements of these biomarkers following AKI diagnosis using sCr. These biomarkers could possibly be utilized clinically to monitor renal function and AKI progression. There is also limited data on these biomarkers beyond the first week of life in premature neonates. Thus, clinical utility after this period remains uncertain.

## Conclusion

As there are several limitations of current AKI diagnostic criteria based on sCr in the neonatal period, biomarkers with greater diagnostic accuracy in this patient demographic are needed to improve patient outcomes. This systematic review and meta-analysis suggests that there are several putative biomarkers for the prediction of AKI in premature infants. In particular, sCysC and uNGAL both show good diagnostic accuracy in this clinical setting. However, evidence is currently only available from a limited number of studies. Of note, > 60% of the studies were conducted in the last 5 years suggesting increased interest in the field, potentially encouraged by enhanced availability of suitable diagnostic tests. To date, many of these biomarkers have only been analysed in a research setting and would need to be scaled for high-throughput analysis before they can be routinely adopted for clinical use. For any of these to be used as a ‘gold standard’ biomarker, they ideally need to have the ability to predict and diagnose AKI and identify the cause, location and type of injury. There is a clinical need to diagnose AKI in premature babies early as this might lead to more appropriate clinical management of these infants with the potential to prevent long-term consequences of AKI [64, 65], especially progression to CKD. Future prospective studies with larger cohorts of participants and appropriate methods to determine adequate diagnostic cut-offs (e.g. Youden’s J statistic) are needed to validate these biomarkers before they can be utilized in clinical practice.

## Supplementary Information

Below is the link to the electronic supplementary material.

Supplementary file 1 (PDF 93 KB)

Supplementary file 2 (PDF 38 KB)

Supplementary file 3 (PDF 103 KB)

Supplementary file 4 (PDF 55 KB)
