# Supplementary material for: Serum and urinary biomarkers to predict acute kidney injury in premature infants: a systematic review and meta-analysis of diagnostic accuracy
Source: J Nephrol. 2022 Apr 6;35(8):2001–14. doi: 10.1007/s40620-022-01307-y (PMC9584850; doi:10.1007/s40620-022-01307-y)
Supplement: Supplementary file 1 — (PDF 93 KB) [file 40620_2022_1307_MOESM1_ESM.pdf]

Article title: Serum and urinary biomarkers to predict acute kidney injury in premature infants: A systematic review and meta-analysis of diagnostic accuracy
Journal name: Journal of Nephrology
Author names: Jenny Kuo, Lisa K Akison, Mark Chatfield, Peter Trnka, Karen M Moritz
Corresponding author: Prof Karen Moritz, School of Biomedical Sciences, The University of Queensland, k.moritz@uq.edu.au

Online Resource 1: Detailed search strategy used for each database.

Database	Search Strategy	No. of Results
CINAHL	((MH "Infant+") OR infant* OR pediatric* OR paediatric* OR neonat* OR newborn OR "new born") AND ((MH "Biological Markers+") OR biomarker* OR marker*) AND ((MH "Kidney Failure, Acute+") OR "acute kidney injury" OR "acute kidney failure" OR "acute renal injury" OR "acute renal failure" OR AKI OR ARF)	203
Cochrane Library	#1 MeSH descriptor: [Infant] explode all trees<br>#2 infant*:ti,ab,kw OR pediatric:ti,ab,kw OR pediatric:ti,ab,kw OR paediatric:ti,ab,kw OR neonat*:ti,ab,kw OR newborn:ti,ab,kw OR "new born":ti,ab,kw<br>#3 #1 or #2<br>#4 MeSH descriptor: [Biomarkers] explode all trees<br>#5 #4 or biomarker*:ti,ab,kw or marker*:ti,ab,kw<br>#6 MeSH descriptor: [Acute Kidney Injury] explode all trees<br>#7 #6 OR "acute renal injury":ti,ab,kw OR "acute renal failure":ti,ab,kw OR "acute kidney injury":ti,ab,kw OR "acute kidney failure":ti,ab,kw OR AKI:ti,ab,kw OR ARF:ti,ab,kw<br>#8 #3 and #5 and #7	42
EMBASE	(“biological marker”/exp OR biomarker*:ab,ti OR marker*:ab,ti) AND (“acute kidney failure”/exp OR “acute renal injury”:ab,ti OR “acute renal failure”:ab,ti OR “acute kidney injury”:ab,ti OR “acute kidney failure”:ab,ti OR aki:ab,ti OR arf:ab,ti) AND (infant/exp OR infant*:ab,ti OR pediatrics/exp OR pediatric:ab,ti OR paediatric:ab,ti OR neonat*:ab,ti OR newborn:ab,ti OR “new born”:ab,ti)	694
PubMed	((infant[MeSH] OR infant*[tiab] OR pediatric[MeSH] OR pediatric[tiab] OR paediatric[tiab] OR neonat*[tiab] OR newborn[tiab] OR "new born"[tiab]) AND (biomarkers[MeSH] OR biomarker*[tiab] OR marker*[tiab]) AND (acute kidney injury[MeSH] OR "acute renal injury"[tiab] OR "acute renal failure"[tiab] OR "acute kidney injury"[tiab] OR "acute kidney failure" OR AKI[tiab] OR ARF[tiab])	526
Scopus	(TITLE-ABS-KEY (infant* OR paediatric* OR pediatric* OR neonat* OR newborn OR "new born")) AND (TITLE-ABS-KEY (biomarker* OR marker*)) AND (TITLE-ABS-KEY (aki OR "acute kidney injury" OR "acute renal injury" OR "acute renal failure" OR "acute kidney failure" OR arf))	653
