# Supplementary material for: Serum and urinary biomarkers to predict acute kidney injury in premature infants: a systematic review and meta-analysis of diagnostic accuracy
Source: J Nephrol. 2022 Apr 6;35(8):2001–14. doi: 10.1007/s40620-022-01307-y (PMC9584850; doi:10.1007/s40620-022-01307-y)
Supplement: Supplementary file 2 — (PDF 38 KB) [file 40620_2022_1307_MOESM2_ESM.pdf]

Article title: Serum and urinary biomarkers to predict acute kidney injury in premature infants: A systematic review and meta-analysis of diagnostic accuracy
Journal name: Journal of Nephrology
Author names: Jenny Kuo, Lisa K Akison, Mark Chatfield, Peter Trnka, Karen M Moritz
Corresponding author: Prof Karen Moritz, School of Biomedical Sciences, The University of Queensland, k.moritz@uq.edu.au

Online Resource 2: Modified 10 item STARD criteria adapted from Coca et al.[1]

Item no.	Validity criterion	Explanation	Scoring
1	Participant recruitment	Was recruitment based on presenting symptoms, results from previous tests, or fact that participants received index tests	Presenting symptoms =1;<br>Previous tests or index tests = 0
2	Participant sampling	Was the study population a convenience sample or a consecutive series?	Consecutive series = 1;<br>Convenience sample = 0
3	Data collection	Was data collection planned before the index test and reference standard were performed prospectively or retrospectively?	Prospective = 1;<br>Retrospective = 0
4	Reference standard	Was the rationale for the reference standard stated?	Stated = 1;<br>Not stated = 0
5	Materials and methods	Were technical specifications of material and methods stated including how and when measurements were taken?	Fully Stated = 2;<br>Partial = 1;<br>Not stated = 0
6	Index Test	Were the definitions of and rationales for the units, cutoffs, and/or categories of the results of the index tests stated?	Stated = 1;<br>Not stated = 0
7	Blinding	Were readers of index test and reference standard blinded to disease state of the participant?	Blinded = 1;<br>Not blinded or not stated = 0
8	Completion	Was the number of participants that did not undergo index tests (no. of tests vs sample size) stated?	Stated=1;<br>[If n numbers match but not stated, still = 1]<br>Not stated = 0
9	Time interval	Was the time interval from index test to reference standard stated?	Stated=1;<br>Not stated = 0

10	Distribution of severity of disease	Was there a representative distribution of severity of disease? (mild, moderate, severe AKI; non-oliguric vs oliguric)	Yes = 1;<br>No = 0;<br>Not stated =0

1. Coca SG, Yalavarthy R, Concato J, Parikh CR. Biomarkers for the diagnosis and risk stratification of acute kidney injury: a systematic review. Kidney Int. 2008 May;73(9):1008-16.
