# Supplementary material for: Serum and urinary biomarkers to predict acute kidney injury in premature infants: a systematic review and meta-analysis of diagnostic accuracy
Source: J Nephrol. 2022 Apr 6;35(8):2001–14. doi: 10.1007/s40620-022-01307-y (PMC9584850; doi:10.1007/s40620-022-01307-y)
Supplement: Supplementary file 3 — (PDF 103 KB) [file 40620_2022_1307_MOESM3_ESM.pdf]

Article title: Serum and urinary biomarkers to predict acute kidney injury in premature infants: A systematic review and meta-analysis of diagnostic accuracy
Journal name: Journal of Nephrology
Author names: Jenny Kuo, Lisa K Akison, Mark Chatfield, Peter Trnka, Karen M Moritz
Corresponding author: Prof Karen Moritz, School of Biomedical Sciences, The University of Queensland, k.moritz@uq.edu.au

Online Resource 3: Diagnostic criteria used for acute kidney injury (AKI).

**Criteria**			
AKIN	**Stage**	**Serum creatinine (SCr[d])**	**Urine output (UO)**
	1	Increase in SCr of ≥0.3 mg/dl (≥26.4 µmol/l) OR increase to ≥150% to 200% from baseline	<0.5 ml/kg/h hour for >6 h
	2	Increase in SCr to >200% to 300% from baseline	<0.5 ml/kg/h for >12 h
	3	Increase in SCr to >300% from baseline (or SCr ≥4.0 mg/dl with an acute increase of >0.5 mg/dl)	<0.3 ml/kg/h for 24 h or anuria for 12 h
KDIGO	**Stage**	**Serum creatinine (SCr)**	**Urine output (UO)**
	1	1.5 – 1.9 times baseline OR ≥0.3 mg/dl increase	<0.5 ml/kg/h for 6-12 h
	2	2.0 – 2.9 times baseline	<0.5 ml/kg/h for ≥12 h
	3	3.0 times baseline OR Increase in SCr to ≥4.0 mg/dl OR Initiation of renal replacement therapy OR In patients <18 years, decrease in eGFR to <35 ml/min/1.73 m^2	<0.3 ml/kg/h for ≥24 h or anuria for ≥12 h
Neonatal KDIGO[a]	**Stage**	**Serum creatinine (SCr)**	**Urine output (UO)**
	0	No change in SCr OR rise <0.3 mg/dL	>0.5 mL/kg/h
	1	SCr rise ≥0.3 mg/dL within 48 h OR SCr rise ≥1.5–1.9 x reference SCr[b] within 7d	<0.5 ml/kg/h for 6-12 h
	2	SCr rise ≥2.0–2.9 x reference SCr[b]	<0.5 ml/kg/h for ≥12 h
	3	SCr rise ≥3 x reference SCr[b] OR SCr ≥2.5 mg/dL[c] OR receipt of dialysis	<0.3 ml/kg/h for ≥24 h or anuria for ≥12 h
Paediatric-modified RIFLE (pRIFLE)	**Stage**	**Estimated Creatinine Clearance (eCCl)**	**Urine output (UO)**
	Risk	eCCl decrease by 25%	<0.5 ml/kg/h for 8 h
	Injury	eCCl decrease by 50%	<0.5 ml/kg/h for 16 h
	Failure	eCCl decrease by 75% OR eCCl <35 ml/min/1.73 m^2	<0.3 ml/kg/h for 24 h or anuric for 12 h
	Loss	Persistent failure >4 weeks	-
	End stage renal disease	End-stage renal disease (persistent failure >3 months)	-

AKIN = Acute Kidney Injury Network[1]; eCCl = estimated creatinine clearance; eGFR = estimated glomerular filtration rate; h = hour; KDIGO = Kidney Disease: Improving Global Outcomes[2-4]; pRIFLE = Paediatric-modified RIFLE (risk, injury, failure, loss, end stage renal disease)[5]; SCr = serum creatinine; UO = urine output

[a] Main differences between neonatal KDIGO and KDIGO criteria is that neonatal KDIGO is based on the lowest previous SCr rather than baseline, stage 1 is a rise within 7 days, and eGFR component is decreased from <35 ml/min/1.73m^2 to <10 mL/min/1.73m^2.
[b] Reference SCr is defined as the lowest previous SCr value.
[c] SCr value of 2.5 mg/dL represents <10 mL/min/1.73m^2.
[d] Conversion factors for units: serum creatinine in mg/dL to μmol/L, ×88.4.

1. Mehta RL, Kellum JA, Shah SV, Molitoris BA, Ronco C, Warnock DG, et al. Acute Kidney Injury Network: report of an initiative to improve outcomes in acute kidney injury. Crit Care. 2007;11(2):R31.
2. Jetton JG, Askenazi DJ. Update on acute kidney injury in the neonate. Curr Opin Pediatr. 2012;24(2):191-96.
3. Kidney Disease: Improving Global Outcomes (KDIGO) Acute Kidney Injury Work Group. KDIGO clinical practice guideline for acute kidney injury. Kidney Int Suppl 2012;2(1):1-138.
4. Jetton JG, Askenazi DJ. Acute kidney injury in the neonate. Clin Perinatol. 2014 Sep;41(3):487-502.
5. Akcan-Arikan A, Zappitelli M, Loftis LL, Washburn KK, Jefferson LS, Goldstein SL. Modified RIFLE criteria in critically ill children with acute kidney injury. Kidney Int. 2007 May;71(10):1028-35.
