# Supplementary material for: Serum and urinary biomarkers to predict acute kidney injury in premature infants: a systematic review and meta-analysis of diagnostic accuracy
Source: J Nephrol. 2022 Apr 6;35(8):2001–14. doi: 10.1007/s40620-022-01307-y (PMC9584850; doi:10.1007/s40620-022-01307-y)
Supplement: Supplementary file 4 — (PDF 55 KB) [file 40620_2022_1307_MOESM4_ESM.pdf]

Article title: Serum and urinary biomarkers to predict acute kidney injury in premature infants: A systematic review and meta-analysis of diagnostic accuracy
Journal name: Journal of Nephrology
Author names: Jenny Kuo, Lisa K Akison, Mark Chatfield, Peter Trnka, Karen M Moritz
Corresponding author: Prof Karen Moritz, School of Biomedical Sciences, The University of Queensland, k.moritz@uq.edu.au

Online Resource 4: Quality assessment criterion scores for each of the included articles.

	Item number										
**Study**	**1**	**2**	**3**	**4**	**5**	**6**	**7**	**8**	**9**	**10**	**Total[a]**
Abdelaal et al 2017	1	1	1	1	2	1	0	1	1	0	**9**
Askenazi et al 2016	1	1	1	1	1	1	0	0	1	0	**7**
Askenazi et al 2011	1	1	1	1	1	1	0	1	1	0	**8**
Elmas et al 2013	1	1	1	1	1	1	0	1	1	0	**8**
El-Gammacy et al 2018	1	1	1	1	1	1	0	1	1	0	**8**
Genc et al 2013	1	1	1	1	2	1	0	1	1	0	**9**
Hanna et al 2016	1	1	1	1	2	1	0	0	1	0	**8**
Ivanisevic et al 2017	1	1	1	1	2	1	0	1	1	0	**9**
Jung et al 2020	1	1	1	1	1	1	0	1	1	0	**8**
Mercier et al 2017	1	1	1	1	1	1	0	1	1	0	**8**
Parravicini et al 2016	1	1	1	1	2	1	0	1	1	0	**9**
Pejovic et al 2015	1	1	1	1	2	1	1	1	1	0	**10**
Sarafidis et al 2014	1	1	1	1	2	1	1	1	1	0	**10**
Tabel et al 2014	1	1	1	1	1	1	0	1	1	0	**8**
Waldherr et al 2019	1	1	1	1	2	0	0	1	1	0	**8**

[a]The total maximum possible score was 11. ≥9 = good; 7-8 = fair; ≤6 = poor quality.
